# Insights From the Current Practice of Pneumococcal Disease Prevention for Diabetic Patients in Saudi Arabia

**DOI:** 10.7759/cureus.23612

**Published:** 2022-03-29

**Authors:** Raed Al-Dahash, Abdallah Kamal, Ashraf Amir, Ashraf Shabaan, Diaa Ewias, Hussam Jnaid, Mussa Almalki, Nabil Najjar, Najlaa Deegy, Saaed Khedr, Samia Bukhary

**Affiliations:** 1 Department of Medicine, Ministry of National Guard-Health Affairs, Riyadh, SAU; 2 Internal Medicine, King Abdullah International Medical Research Center, Riyadh, SAU; 3 Internal Medicine, King Saud bin Abdulaziz University for Health Sciences, Riyadh, SAU; 4 Internal Medicine and Endocrinology/Diabetes Care, Dallah Hospital, Riyadh, SAU; 5 Family Medicine, International Medical City, Jeddah, SAU; 6 Diabetes Control Center, Ghassan Najib Pharaon (GNP) Hospital, Jeddah, Jeddah, SAU; 7 Internal Medicine, Saudi German Hospital, Jeddah, SAU; 8 Family Medicine, King Faisal Specialist Hospital and Research Center, Riyadh, SAU; 9 Obesity, Endocrine, and Metabolism Center, King Fahad Medical City, Riyadh, SAU; 10 Internal Medicine, Diabetes Center, Hai Al Jamea Hospital, Jeddah, SAU; 11 Internal Medicine Department, Qassim University, Qassim, SAU; 12 Endocrinology and Diabetes, Diabetes Center, Alhabib Medical Group Hospitals, Riyadh, SAU; 13 Endocrinology and Diabetes, King Fahad Hospital, Riyadh, SAU

**Keywords:** types 2 diabetes, local vaccination guidelines, pneumococcal vaccine, pneumonia, diabetes

## Abstract

Pneumonia is the most frequent cause of hospitalization, resulting in a high risk of mortality. Diabetic patients are at high risk of aquatinting pneumococcal infections with their consequent complications. Despite the fact that glycemic control of the patients reduces the risk of diabetic complications and enhances their immunity, pneumococcal vaccination should still be given irrespective of the patients’ glycemic control. The purpose of this review is to address the present situation of pneumococcal disease prevention in diabetic patients in the Kingdom of Saudi Arabia (KSA) and to gather professional recommendations to overcome the vaccination-related barriers. Onsite insights of scientific leaders in family medicine, endocrinology, and internal medicine in Riyadh and Jeddah were gathered and linked with the available literature to tackle the current practice of pneumococcal disease prevention in diabetic patients in the Kingdom of Saudi Arabia. Pneumococcal vaccination importance is still not well recognized among endocrinologists across the Kingdom of Saudi Arabia, despite the availability of established local recommendations and the National Immunization Program. The prevention of serious and fatal pneumococcal diseases should be one of the treatment pillars for diabetic patients, and it is not less important than controlling other risk factors.

## Introduction and background

Diabetes is a complex, progressive, and heterogeneous disease associated with a significant burden in terms of treatment cost, management of disease complications, disability, and loss of productivity [1-3]. The burden of diabetes is expanding in the Gulf Cooperation Council (GCC) region [3-5].

Diabetes represents a global public health emergency. The prevalence of diabetes in the Middle East and North African (MENA) region is high [4]. The prevalence of diabetes in the Arab world is expected to increase by 96.2% in 2035 [3]. In 2017, the Kingdom of Saudi Arabia (KSA) had the highest age-adjusted diabetes prevalence (17.7%) [5]. The worldwide prevalence of diabetes among subjects over 65 years was 123 million in 2017, a number that is expected to double in 2045 [6].

Diabetes increases mortality rates and healthcare expenditures. As per the International Diabetes Federation (IDF), in 2019, diabetes and its complications were responsible for an estimated 418,900 deaths in adults aged 20-79 years in the MENA region. Moreover, health expenditure due to diabetes reached $24.9 billion [6]. Diabetes mellitus is associated with several complications that ultimately impact the overall survival of these patients. Diabetic patients are more susceptible to unusual pathogenic infections, such as pneumonia [7-9].

Although adequate resources and facilities are established and provided by the Saudi government to offer the optimum management of diabetes, the disease burden and the other barriers related to the unorganized healthcare system are considered the primary significant reasons behind uncontrolled diabetes.

In this review, we reported the proceedings of two scientific advisory boards that took place in Riyadh and Jeddah in 2017 and reviewed the available literature tackling the current practice of pneumococcal disease prevention in diabetic patients. A total of 13 scientific experts specializing in family medicine, endocrinology, and internal medicine were invited to discuss the local guidelines for pneumococcal disease immunization and their implementation in the Saudi health sector. The experts shared their insights regarding the local epidemiology of diabetes and the impact of pneumonia on diabetic patients. They also specified the specialties responsible for diabetic patients’ vaccination and elaborated on diabetic patients’ vaccination journey.

## Review

The burden of T2DM in KSA

Based on the advisors’ discussion, in KSA, the mean age of patients with type 2 diabetes mellitus (T2DM) is 59.3 years. The majority of diabetic patients suffering from pneumonia are referred to public institutions instead of the private sector. The percentage of overall T2DM patients hospitalized due to pneumonia is estimated to be 16-25% in Jeddah and 6-15% in Riyadh. It is roughly estimated that <5% of immunized T2DM patients are hospitalized due to pneumonia, and <5% of hospitalized T2DM patients’ deaths are related to pneumonia.

Hyperglycemia and infections

T2DM is the most common type of diabetes in the GCC region, specifically KSA [3]. Diabetes is recognized as an immunosuppressive disease. Many mechanisms explain the association between hyperglycemia and immunosuppression [7].

Hyperglycemia affects both humoral and cellular immunity as it provokes oxidative stress, causing the immune system's exhaustion and inflammatory cytokines release, exposes the internal organs to an extra amount of glucose, and provides nutrients to foreign organisms (such as bacteria or viruses). These factors render patients more vulnerable to infections [7,9,10]. Said infections are difficult to treat and cause more inflammation leading to a hyperglycemic state [7].

Diabetes negatively affects the micro-and macro-vasculature of various organs, rendering them exposed to very poor blood supply and increasing their vulnerability to infections. Hyperglycemia results in immune suppression, which in its turn increases the risk of infections [7].

Pneumococcal disease in diabetic patients

Diabetes is sought to be an independent risk factor for increased susceptibility to acquiring bacterial infections, such as pneumonia [9,11]. Diabetic patients older than 18 years are up to three times more likely to contract pneumonia when compared with healthy people of the same age group, while diabetic patients aged >65 years are up to three times more likely to contract pneumonia compared with younger patients [12]. Pneumonia is the second most common cause of hospitalization in diabetic patients. The incidence of hospitalization due to pneumonia is three times higher in adult diabetic patients compared with healthy adults [13]. Streptococcus pneumonia is among the most commonly identified pathogens for pneumonia in diabetic patients [14].

Due to the chronic nature of diabetes, diabetic patients are at high risk of contracting pneumococcal diseases in all seasons - not only in winter - unlike seasonal influenza [15]. The outbreak of an infectious disease highly increases in crowded seasons, such as Ramadan and Hajj [16]. This explains why most experts recommend that the most appropriate time to discuss the importance of vaccination with patients is before Hajj/Umrah.

The risk of pneumonia in diabetic patients increases with the number of comorbidities. Comorbidities include asthma, chronic kidney disease (CKD), cardiovascular diseases (CVD), cataracts, retinopathy, and neuropathy [12].

The risk of mortality in diabetic patients increases after hospitalization due to pneumonia [16]. It was reported that multiple deaths in the intensive care unit (ICU) were due to chest infections in diabetic patients with comorbidities, such as stroke and cardiovascular disease. The risk of mortality due to community-acquired pneumonia (CAP) after hospitalization is significantly higher in diabetic patients compared with non-diabetic patients [17].

Many pneumonia infections are resistant to antibiotics, and accordingly, they could be fatal [18]. Pneumonia is considered the leading cause of hospital admissions [19]. Shirah et al. concluded that the mortality rates of pneumonia cases admitted to the ICU were approximately 21%, which aligns with the percentage of hospitalized patients in Jeddah and Riyadh. The most common at-risk conditions include chronic obstructive pulmonary disease (COPD), bronchial asthma, diabetes mellitus, and chronic heart failure [16].

Immunization in KSA

As per the experts, immunization is not a common practice among some local clinics. Healthcare professionals (HCPs) recommend pneumococcal immunizations, while a minority of diabetic patients seek immunizations without guidance. In Jeddah, endocrinologists are the principal physicians responsible for prescribing the pneumococcal vaccine for T2DM patients since they have full access to prescribe the pneumococcal vaccine for T2DM patients. Whereas in Riyadh, family physicians are primarily responsible for the pneumococcal vaccine prescription for T2DM patients. Referral to nephrologists is possible according to the results of renal function tests.

According to the panel, the pneumococcal vaccine can be administered in a timely manner with either diagnosis, treatment initiation, or one of the follow-up visits (20-30% of diabetic patients). However, it is argued that it might be unwise to discuss the importance of vaccination with patients who are still in the diagnosis or treatment initiation phases. All the advisors agreed that the most appropriate time to discuss the importance of immunization with patients is before Hajj/Umrah. In KSA, the pneumococcal vaccine is not mandatory prior to Hajj, unlike the meningococcal vaccine.

The ideal time to seek pneumococcal immunization was believed to be zero to six months from diagnosis, based on the presence of the associated comorbidities and risk factors. The vaccination step could be considered after six months of diagnosis to allow for time to adjust the medications and the glycemic state of the patient.

Although the glycemic control of the patients reduces the risk of diabetic complications and enhances the immunity of the patients, pneumococcal vaccination should be given irrespective of the patients’ glycemic control. It is important to encourage diabetic patients to receive three main vaccinations against influenza, hepatitis, and pneumococcal infections.

As per the experts’ risk stratification, diabetic patients could be classified into three categories: low-risk, intermediate-risk, and high-risk patients. Diabetic patients with high-risk comorbidities such as heart failure, pulmonary diseases, hypertension, renal impairment, and old age are categorized as high-risk patients and should receive immediate pneumococcal vaccination.

Many years ago, it was very difficult to convince people about the significance of vaccinations in general and for adults specifically. In the meantime, it is much easier to administer pneumococcal vaccines to diabetic patients. Raising public awareness about adult pneumococcal vaccination and optimizing the healthcare system for diabetic patients is crucial. Different domains, including the concept of adult vaccination, smoking cessation, and the awareness of risk factors, should be comprehensively addressed by the healthcare authorities and pharmaceutical companies in order to achieve state-of-the-art management of diabetes.

The leading barriers to refusing pneumococcal immunization in T2DM patients

Awareness among patients plays an essential role in deciding whether to receive the vaccination or not. The role of the media in raising awareness of public health issues, including the diabetic population, to protect them against pneumonia, has a strong impact.

The most common barriers to vaccination are accessibility, availability, and the fear of potential side effects of vaccines; lack of established recommendations from the Ministry of Health (MOH); lack of knowledge about the vaccine; fear of injection site pain; and lack of time for the physicians to recommend vaccination. Many strategies could be adopted to overcome such barriers (Table 1).

**Table 1:** Professional recommendations for the management of the vaccination patients-related barriers HCPs: health care professionals

Barrier	Recommendations
1. Lack of knowledge	Raising the public and HCPs awareness
2. Unavailability of the vaccine in some clinics	Providing vaccines
3. Fear of potential side effects of the vaccine	Correcting misbeliefs by offering appropriate patient education
4. Unwillingness of the patients to receive vaccination	Educating patients about the safety and protective effects of vaccines, and educating patients about the mortality risk of the disease

Optimization of pneumococcal vaccination in T2DM patients

Strategies that could be adopted to optimize vaccination include raising awareness through establishing educational flyers and guidelines and cascading the knowledge from endocrinologists to GPs and patient educators.

Raising the awareness of insurance companies about the risk of pneumococcal disease in diabetic patients is highly impactful. Adopting preventive medicine, different screening tools, and vaccination have an important role in reducing the economic burden. The easier and smoother flow of institutional procedures, the availability of the vaccines at reasonable prices, and convincing insurance companies about the protective effects of the pneumococcal vaccines, along with the burden on patients if they are left unvaccinated, are the main points of concern to raise awareness about pneumococcal vaccination. The key performance indicators (KPIs) of pneumococcal vaccination should be considered to the same extent as those exhibited in target glycated hemoglobin and blood pressure in diabetes. Raising the awareness of endocrinologists about pneumococcal vaccination is critically important.

It is difficult to identify the vaccination status of diabetic patients who were hospitalized due to the development of pneumococcal infections. Accordingly, the mortality rate for this category is not known.

Pneumococcal vaccination's importance is not well recognized yet among endocrinologists across KSA. Accordingly, pneumococcal vaccination urgency could be considered a risk given the fact that most patients are not fully convinced of the necessity of receiving the influenza vaccine in the first place. The four leading accessibility barriers are institutions' infrastructure, inclusion procedures, medical facility regulations, and insurance policies (Table 2).

**Table 2:** Vaccine accessibility barriers

Leading accessibility barriers	Suggested recommendations
Institution’s infrastructure	Build a room facilitated for the vaccination procedures
	Increase human resources
	Provide vaccines in all centers
Inclusion procedure	Formulate clearly stated and well-established regulations
	Decrease the restriction of vaccination to only some of the populations
	Establish pathways to include vaccination as a part of diabetes management
	Authorize physicians to order the vaccine
Medical facilities regulations	Provide vaccines
	Launch small campaigns specified to each institution to increase awareness
	Shorten the vaccination processes held by the vaccination committee
Insurance policies	Expand the coverage by insurance to all patients

Pneumococcal disease immunization for T2DM patients

Although the American Diabetes Association (ADA) recommends pneumococcal immunization for diabetic patients, especially the elderly population [19], the concept of immunization is undervalued by some practitioners and institutions in KSA. The experts recommended formulating local guidelines across different hospitals in KSA to raise awareness about pneumococcal vaccinations for diabetic patients.

A well-structured patient pathway starts at the clinics of general practitioners (GPs) or endocrinologists in collaboration with patient educators, followed by a referral to other specialties (nephrologists) as needed. Such a pathway would be highly beneficial in facilitating the introduction of the vaccine to patients.

In KSA, the Saudi Thoracic Society recommends pneumococcal vaccination for all pilgrims to Hajj [21]. The Saudi MOH had established key performance indicators and provided vaccines for pneumococcal immunization in diabetic centers [21]. Pneumococcal vaccination must be considered an obligatory program for the diabetic population, which is similar to the cases of hepatitis and polioviruses.

Merging pneumococcal vaccination awareness campaigns with influenza vaccine campaigns could lead to a higher percentage of vaccinated patients. Convincing patients to follow the preventive vaccination approach could be easily endorsed by the long-term mutual trust between patients and their physicians.

Local vaccination guidelines

International guidelines recommend the administration of influenza and pneumococcal vaccinations for diabetic patients. Recommendations for pneumococcal vaccination in diabetic patients might vary across guidelines depending on the vaccine type, the patient's age at the time of vaccination, and the dosing schedule [22]. Pneumococcal vaccines, such as the 13-valent pneumococcal conjugate vaccine (PCV13), are currently licensed for the prevention of pneumococcal disease in diabetic patients [19,21-23]. PCV13 covers 50-70% of invasive pneumococcal disease cases [19,24]. PCV23 exhibits additional serotype coverage without memory cell formation, resulting in the need for dose repetition. More protection against the disease is achieved when the broader serotypes are vaccinated [25].

The local adult National Immunization Program (NIP) recommended that the dose of PCV be one dose for adults with comorbid/immunocompromised conditions and adults aged 65 years or older [26,27]. The Saudi Thoracic Society guidelines recommend pneumococcal vaccination for all children <5 years old, adults ≥50 years old, and people ≥6 years old with certain risk factors. These recommendations are based on the presence of a large number of comorbidities in the KSA population >50 years of age, many of whom have risk factors for contracting pneumococcal infections [21].

In 2016, the Saudi Thoracic Society Pneumococcal Vaccination Guidelines announced their pneumococcal vaccination recommendations (Table 3) [20,21].

**Table 3:** The Saudi Thoracic Society Pneumococcal Vaccination Guidelines 2016 *In case of CSF leak and cochlear implant give PCV13 followed by one dose of PPSV23 ≥8 weeks later [21]. **In case of CSF leak and cochlear implant give a single dose of PCV13 first, followed ≥8 weeks later by a dose of PPSV23. CSF: cerebrospinal fluid; PCV13: 13-valent pneumococcal conjugate vaccine; PPSV23: 23-valent pneumococcal polysaccharide vaccine.

Recommended pneumococcal vaccination for high-risk individuals aged >6 years and <50 years	Conditions	Not previously vaccinated	Already received PPSV23 only
	High-risk immunocompetent (patients who have the ability to produce a normal immune response) [20]	Administer one dose of PPSV23*	No need to repeat unless age is >50 years**
	Immunocompromised (patients who have a weakened immune system) [20]	Give a single PCV13 dose first, followed ≥8 weeks later by a dose of PPSV23. Revaccination with PPSV23 every 5 years.	Give single PCV13 dose first, followed ≥8 weeks later by a dose of PPSV23 (at least 5 years from last PPSV23 dose). Revaccination with PPSV23 every 5 years.
	Functional and anatomical asplenia		

## Conclusions

Diabetic patients are vulnerable to unusual types of pathogenic infections. Therefore, implementing an immunization program for diabetics should be an obligation. A well-structured patient pathway starts at the clinics of GPs or endocrinologists in collaboration with patient educators, followed by a referral to other specialties (nephrologists) as needed. Such a pathway would be highly beneficial in facilitating the introduction of vaccination to patients. It is recommended to formulate local guidelines in KSA to raise awareness about pneumococcal vaccinations for diabetic patients. Pneumococcal vaccination should be given regardless of the patient's glycemic control. Convincing patients to follow the preventive vaccination approach could be easily endorsed by the long-term mutual trust between patients and their physicians. Similar to glycated hemoglobin and blood pressure targets in diabetes, the key performance indicators of pneumococcal vaccination should be considered. Further studies could be conducted to investigate the effects of education and awareness among the population on the vaccination rate.
